# Artificial Intelligence to Analyze the Cortical Thickness Through Age

**DOI:** 10.3389/frai.2021.549255

**Published:** 2021-10-13

**Authors:** Sergio Ledesma, Mario-Alberto Ibarra-Manzano, Dora-Luz Almanza-Ojeda, Pascal Fallavollita, Jason Steffener

**Affiliations:** ^1^ Faculty of Health Sciences, University of Ottawa, Ottawa, ON, Canada; ^2^ School of Engineering, University of Guanajuato, Guanajuato, Mexico

**Keywords:** modeling, cortical thickness, artificial neural network, derivative, changes with age, adaptive models, neuroimaging

## Abstract

In this study, Artificial Intelligence was used to analyze a dataset containing the cortical thickness from 1,100 healthy individuals. This dataset had the cortical thickness from 31 regions in the left hemisphere of the brain as well as from 31 regions in the right hemisphere. Then, 62 artificial neural networks were trained and validated to estimate the number of neurons in the hidden layer. These neural networks were used to create a model for the cortical thickness through age for each region in the brain. Using the artificial neural networks and kernels with seven points, numerical differentiation was used to compute the derivative of the cortical thickness with respect to age. The derivative was computed to estimate the cortical thickness speed. Finally, color bands were created for each region in the brain to identify a positive derivative, that is, a part of life with an increase in cortical thickness. Likewise, the color bands were used to identify a negative derivative, that is, a lifetime period with a cortical thickness reduction. Regions of the brain with similar derivatives were organized and displayed in clusters. Computer simulations showed that some regions exhibit abrupt changes in cortical thickness at specific periods of life. The simulations also illustrated that some regions in the left hemisphere do not follow the pattern of the same region in the right hemisphere. Finally, it was concluded that each region in the brain must be dynamically modeled. One advantage of using artificial neural networks is that they can learn and model non-linear and complex relationships. Also, artificial neural networks are immune to noise in the samples and can handle unseen data. That is, the models based on artificial neural networks can predict the behavior of samples that were not used for training. Furthermore, several studies have shown that artificial neural networks are capable of deriving information from imprecise data. Because of these advantages, the results obtained in this study by the artificial neural networks provide valuable information to analyze and model the cortical thickness.

## 1 Background

In the last few years, machine learning techniques have been used in common applications (Alpaydin, 2016). In this paper, we use one technique from Artificial Intelligence to analyze the progress of the cortical thickness with age. This study includes data from 1,100 healthy individuals. The cortical thickness was measured using FreeSurfer which is a fully automated software for measuring several parameters in the brain including neuroanatomic volume and cortical thickness (McCarthy et al., 2015).

Several studies illustrate the relevance of the analysis of the cortical thickness through the life span. For instance, the authors in (Steffener et al., 2016) indicate that brain aging can be analyzed taking into consideration the inevitable and universal effects of advancing age and the effects resulting from a lifetime of exposures. These effects and a decreased cortical thickness in some regions of the brain may be related to some mental disorders or cognitive decline (Fouche et al., 2017; Razlighi et al., 2017). Thus, some studies have indicated correlations between disease states and cortical thickness, see the references in (Scott et al., 2009).

In the state of the art, there are many studies about the modeling of changes in the cortical thickness. The authors in (Scott et al., 2009) propose a voxel-based method to measure the cortical thickness utilizing inversion recovery anatomical magnetic resonance images. Churchwell et al. use separate hierarchical multiple regressions to analyze changes with age in the cortex thickness in specific zones in the brain (Churchwell and Yurgelun-Todd, 2013). Additionally, it has been suggested that brain aging is a process influenced by degenerative and restorative activities (Fjell et al., 2014). Consequently, the resulting process can be linear and non-linear. Similarly, it has been proposed that cortical thickness changes follow non-linear patterns across childhood and adolescence, and these changes vary to some degree by cortical region (Wierenga et al., 2014; Piccolo et al., 2016; Sowell et al., 2007).

In this sense, the thinning of the cortical thickness has been analyzed. For instance, Tamnes et al. describe the age-related changes in cortical thickness, their findings revealed regional age-related cortical thinning (Tamnes et al., 2010), see also (Salat et al., 2004). The authors in (McGinnis et al., 2011) analyze the thinning of the cerebral cortex in different regions of the brain in the course of aging. Chen et al. demonstrate age-related alterations in the modular organization of the human brain structural networks using regional cortical thickness measurements (Chen et al., 2011). Lemaitre et al. use linear regressions of age, their studies indicate an associated global age-related reduction in cortical thickness, surface area and volume (Lemaitre et al., 2012). On the other hand, it has been indicated that cortical surface area is an increasingly used brain morphology metric that is ontogenetically and phylogenetically distinct from the cortical thickness and offers a separate index of neuro-development and disease (Winkler et al., 2018).

## 2 Artificial Neural Networks

An artificial neural network is a computational technique motivated by a specific behavior found in the brain (Marsland, 2015). A neural network is composed of basic units of processing called neurons. Inside the network, the neurons are organized in layers. Artificial neural networks are used for: image classification, image processing, signal processing, prediction, pattern recognition, function approximation, and other applications (Jin et al., 2017; Jordan and Mitchell, 2015). From a practical point of view, artificial neural networks can be used to create a model using only a set of data samples (Russell and Norvig, 2020; Masters, 2015). The main advantage of using an artificial neural network to model the cortical thickness is that the network creates the model that best fits the patterns in the data. In other words, an artificial neural network is capable of learning and modeling non-linear and complex relationships. Additionally, the neural network is immune to noise in the data samples and can infer unseen relationships on unseen data. Therefore, the models obtained are able to generalize and predict on unseen data. Furthermore, research has shown that artificial neural networks have a great capability of deriving information from complex or imprecise data.

## 3 Dataset Description

The simulations in this study were performed using a dataset with information from approximately 1,100 healthy individuals. This dataset was built by combining data from four different common datasets: IXI, MMRR, NKI, and OASIS. Table 1 includes a sample from one patient of the cortical thickness for each dataset. These datasets are briefly discussed next.

**TABLE 1:** Cortical thickness in millimeters from one person in each database.

Database	IXI		MMRR		NKI		OASIS	
Age (years)	39		25		41		74	
	**Left**	**Right**	**Left**	**Right**	**Left**	**Right**	**Left**	**Right**
	Caudal anterior cingulate	2.432	2.395	2.981	3.201	2.344	2.545	2.7	2.694
Caudal middle frontal	2.23	2.326	2.634	2.578	2.516	2.422	2.351	2.413
Cuneus	1.895	1.663	1.918	1.761	1.935	1.874	1.682	1.805
Entorhinal	3.356	3.728	4.093	3.868	2.808	2.958	2.876	3.053
Fusiform	2.486	2.558	2.657	2.67	2.457	2.538	2.274	2.199
Inferior parietal	2.426	2.356	2.307	2.303	2.338	2.413	2.221	2.267
Inferior temporal	2.892	2.751	2.777	2.832	2.509	2.519	2.57	2.205
Isthmus cingulate	2.214	2.086	2.702	2.38	2.222	2.356	2.031	2.35
Lateral occipital	2.017	2.097	1.863	1.962	2.005	2.066	2.085	2.001
Lateral orbitofrontal	2.522	2.795	3.085	2.95	2.679	2.497	2.538	2.604
Lingual	1.774	1.762	2.096	2.086	1.961	1.911	1.784	1.837
Medial orbitofrontal	2.53	2.444	2.701	2.628	2.633	2.414	2.159	2.553
Middle temporal	2.856	2.825	2.792	2.845	2.607	2.716	2.561	2.548
Parahippocampal	2.456	2.509	3.339	3.143	2.787	2.608	2.035	2.496
Paracentral	2.108	2	2.579	2.395	2.209	2.253	2.214	2.136
Pars opercularis	2.665	2.307	2.69	2.768	2.549	2.635	2.456	2.528
Pars orbitalis	2.464	2.529	2.893	2.771	2.45	2.332	2.308	2.612
Pars triangularis	2.243	2.4	2.533	2.431	2.287	2.364	2.077	2.243
Pericalcarine	1.441	1.308	1.528	1.642	1.58	1.554	1.482	1.454
Postcentral	1.98	1.901	2.349	2.262	2.144	2.127	2.094	2.039
Posterior cingulate	2.397	2.311	2.79	2.655	2.282	2.229	2.234	2.432
Precentral	2.28	2.339	2.248	2.344	2.574	2.449	2.317	2.231
Precuneus	2.28	2.231	2.625	2.438	2.309	2.22	2.285	2.126
Rostral anterior cingulate	2.69	2.899	3.118	3.406	2.894	2.531	3.041	2.908
Rostral middle frontal	2.224	2.34	2.383	2.356	2.373	2.266	2.283	2.15
Superior frontal	2.558	2.565	2.674	2.812	2.518	2.483	2.592	2.477
Superior parietal	2.135	1.978	2.198	2.084	2.311	2.201	2.128	2.168
Superior temporal	2.774	2.826	2.824	3.023	2.771	2.774	2.614	2.633
Supramarginal	2.482	2.414	2.577	2.577	2.545	2.478	2.302	2.309
Transverse temporal	1.893	1.968	2.713	2.628	2.332	2.364	2.621	2.285
Insula	3.072	2.749	3.169	3.242	3.01	2.915	2.942	3.049

### 3.1 IXI Dataset

This dataset contains approximately 600 magnetic resonance images from normal and good health individuals. The data was collected at three different hospitals in London: Hammersmith hospital, Guy’s hospital and the Institute of Psychiatry. The IXI dataset was prepared during the project called Information eXtraction from Images, (Information eXtraction from Images, 2019).

### 3.2 MMRR Dataset

The Multi-Modal MRI Reproducibility Resource dataset was built using information from 21 healthy volunteers. In the MMRR dataset, all volunteers did not have a history of neurological conditions, and therefore, all of them were used in this study. This dataset has 42 records and each record includes information from a 1-h scan session (Landman et al., 2011).

### 3.3 NKl Dataset

The Nathan Klein Institute - Rockland Sample (NKI-RS) is an attempt to create a large-scale community sample. This dataset includes data from different types of assessments including advanced neuroimaging. The dataset has 186 T1-weighted images from 99 males and 87 females.

### 3.4 OASIS Dataset

The Open Access Series of Imaging Studies dataset is a set of magnetic resonance images collected from 416 individuals between the ages of 18–96 years (Marcus et al., 2007). This dataset is public and can be used for research. As this study focuses only on healthy individuals, data coming from patients with a mental disease was discarded, and therefore, not used. Consequently, data from only 313 individuals were used for the computer simulations and analysis performed in this work.

## 4 Methodology

In this study, the cortical thickness of the images provided in (Tustison et al., 2014) was used for the training and validation of 62 artificial neural networks. The total number of records in this dataset was approximately 1,100. Each record had the sex and age of each individual. Additionally, each record included the values of the cortical thickness in 31 regions in the left hemisphere of the brain and 31 regions in the right hemisphere, see Fischl (2012) and Klein and Tourville (2012).

To create the neural network models, several steps were performed. First, the input data, the age of each person in the dataset, was linearly scaled so that all the values at the input of the network were in the range of −1 to 1. Second, the cortical thickness values were also scaled using a linear transformation so that all target values at the output of the network were in the range of −1 to 1. Third, each neural network was trained in two steps. In the first step, a non-greedy optimization method called simulated annealing was used to find initial values of the weights connecting the neurons in the network. Then, a gradient-based method was used to quickly optimize the values of the weights by moving the weights in the opposite direction of the gradient of the error. Once the networks were trained, we validated the performance of the network by measuring the mean squared error between the predicted value and the observed data from the validation set.

### 4.1 Training and Validation of the Artificial Neural Networks

**FIGURE 1:**
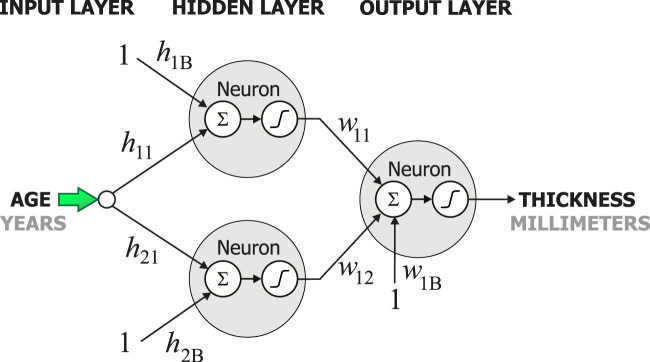
Structure of the artificial neural network used to model the cortical thickness.

Once the dataset was ready, 62 multilayer neural networks were created using the Neural Lab software (Ledesma et al., 2017). All 62 networks had three layers: the input layer, the hidden layer, and the output layer as shown in Figure 1. All neurons in the network were designed to use the hyperbolic tangent as their activation functions. The neurons were connected with weights, these are denoted by *h* and *w* in Figure 1. Each network had one input, the age, and one output, the cortical thickness of one specific region of the brain as in Figure 1. Thus, each neural network had one neuron in the output layer. The number of neurons in the hidden layer was iteratively determined as follows. First, the complete dataset with the 1,100 cases was split into two datasets: the training set and the validation set. Second, each network was trained with zero neurons in the hidden layer. Both the mean squared error for training and the mean squared error for validation were computed. Then, the number of neurons in the hidden layer was increased by one. Again, the mean squared error for training and the mean squared for validation were computed. This iterative process was stopped when the mean squared error during validation did not decrease. The main conclusion obtained from this iterative process was that only two neurons in the hidden layer were necessary to model the cortical thickness.

In this case, 80% of the cases were included in the training set, and the 20% remaining cases were used to build the validation set. The training of the 62 artificial neural networks was performed in two steps using the parameters shown in Table 2. The training of each neural network began using simulated annealing. Then, the method of Levenberg–Marquardt was used to improve the training.

**TABLE 2:** Methods and parameters used for training.

Simulated annealing
Initial temperature	15
Final temperature	0.001
Number of temperatures	100
Iterations per temperature	100
Cooling schedule	Linear
Levenberg-Marquardt	
Number of iterations	1,000
Goal (mean squared error)	1 × 10^–5^

### 4.2 Derivative Computation

In the field of numerical differentiation, there are some methods to estimate the numerical value of the derivative of a function. One common method to approximate the derivative of a function is based on finite differences. There are three types of differences: forward difference, backward difference, and central difference. These differences are associated with a stencil or kernel. A stencil s (or kernel) is a set of *N* points that are arranged in the vicinity of a point of interest (Hassan et al., 2012). For instance, the stencil

s=[−1,0,1] (1)

is used to describe a stencil with three points (*N* = 3) in the vicinity of the point of interest. The numbers in the stencil indicate the time steps, 0 represents the current value, − 1 represents the previous value, and 1 represents the next value. In general, a stencil with *N* points is represented as

$$s=\left[s_{1},s_{2},s_{3},\cdots s_{N}\right].$$ (2)

For instance, when *N* = 5, the derivative is computed using five points in the vicinity of the point of interest. Consequently, when the value of *N* is increased, the accuracy of the derivative also increases. However, when working in the upper or lower ends of the data, it is important to use different stencils to compute the derivative for each point. That is, the point of interest must be dynamically located inside the stencil to compensate for the missing data, see (Hassan et al., 2012). For the stencil s in Equation 2, the finite difference coefficients *c*_1_, *c*_2_, ⋯, *c*_*N*_, can be obtained by solving the system of linear equations

$$\left(\begin{array}{cccc} {\left(s_{1}\right)}^{0} & {\left(s_{2}\right)}^{0} & \cdots & {\left(s_{N}\right)}^{0}\\ {\left(s_{1}\right)}^{1} & {\left(s_{2}\right)}^{1} & \cdots & {\left(s_{N}\right)}^{1}\\ \vdots & \vdots & \ddots & \vdots \\ {\left(s_{1}\right)}^{N-1} & {\left(s_{2}\right)}^{N-1} & \cdots & {\left(s_{N}\right)}^{N-1} \end{array}\right)\left(\begin{array}{c} c_{1}\\ c_{2}\\ \vdots \\ c_{N} \end{array}\right)=d!\left(\begin{array}{c} \delta_{0,d}\\ \delta_{1,d}\\ \vdots \\ \delta_{N-1,d} \end{array}\right)$$ (3)

where *d* is the order of derivative and *δ*_*i*,*j*_ is the Kronecker delta, see (Hassan et al., 2012). The main advantage of using this method is that different stencils can be used to estimate the derivative at different points of interest increasing the accuracy of the computation. It is important to note that Equation 3 cannot be used to estimate the derivative in a non-differentiable region. However, as it can be seen from databases in the state of the art, changes in the cortical thickness are slow and non-differentiable regions were not found in the four databases used in this study.

## 5 Computer Simulations and Results

The computer simulations performed in Section 4.1 were used to determine the proper number of neurons in the hidden layer and to validate the performance of the models. However, once the validation process was finished, it was convenient to create new models by performing the training of the networks using all samples in the data set. Therefore, all the 62 artificial neural networks were again trained, but in this case, all the 1,100 cases (instead of only 80% of the cases) were used. The training was performed as before using the parameters in Table 2. According to the results of the computer simulations performed in Section 4.1, all neural networks had two neurons in the hidden layer.

### 5.1 Cortical Thickness Progress With Age

As it is well known, artificial neural networks may be used to create a model when there is not a mathematical equation to represent the data (Kelleher et al., 2015; Goodfellow et al., 2016). In this study, artificial neural networks were used to model the changes in cortical thickness in the brain at different ages. Specifically, for each region in the brain, one artificial neural network was used to model the cortical thickness in that region. Thus, a total of 62 artificial neural networks were trained and validated to model the cortical thickness of the brain. There are several approaches that can be used to model the different regions of the brain. For instance, instead of using 62 neural networks, it is possible to design a single neural network with 62 outputs. However, computer simulations showed that the performance of the single neural network was very similar to the performance of the 62 neural networks.

The results of the computer simulations indicated that the mean squared error during the training of the artificial neural networks was from 0.016 to 0.031. During the validation of the models, the computer simulations indicated that the variations between the observed data and the predicted results had errors from 0.016 to 0.033. Finally, to build the models, a new set of artificial neural networks was trained using the whole dataset. In this case, the mean squared error was in the range of 0.017–0.034. To our knowledge, this is the first study to use this type of approach to analyze changes in the cortical thickness.

To ease the presentation of the computer simulations, the models obtained by the artificial neural networks were organized manually in clusters. In this sense, each cluster included those regions which exhibit similar behavior through age. A total of six clusters were created based on the patterns observed in the cortical thickness. We chose this number of clusters because most of the patterns observed in the 62 regions of the brain were represented using only six clusters. However, it is important to mention that if more clusters are used, each cluster will include very few regions. These clusters are described next.

#### 5.1.1 Changes in Cortical Thickness Around 25 years of Age

**FIGURE 2:**
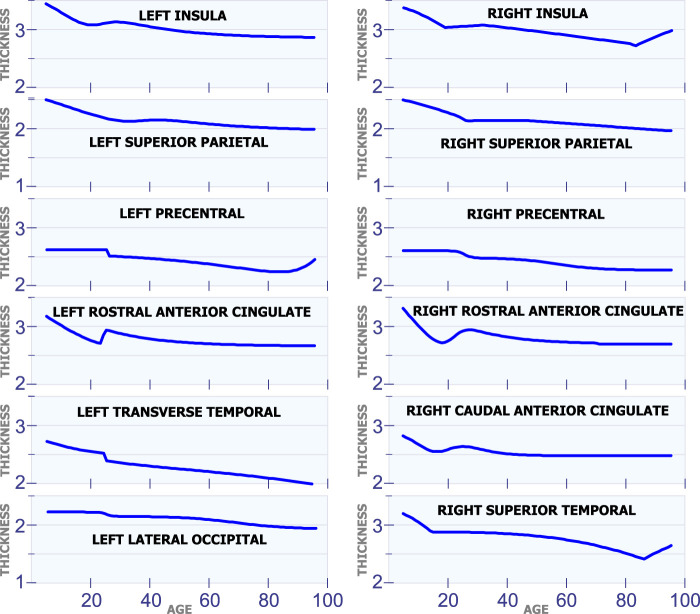
Regions with changes in cortical thickness around 25 years of age.

**FIGURE 3:**
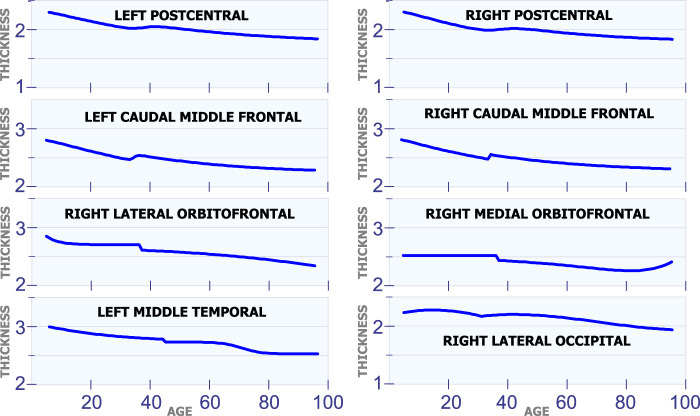
Regions with changes in cortical thickness around 40 years of age.

**FIGURE 4:**
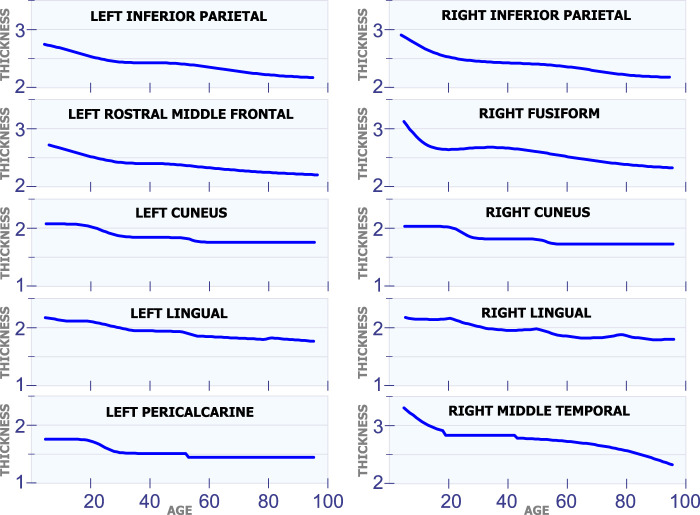
Regions with changes in cortical thickness around 50 years of age.

**FIGURE 5:**
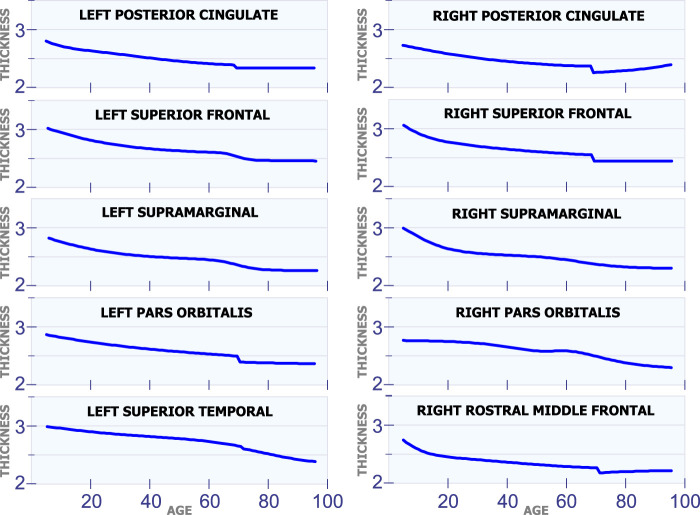
Regions with changes in cortical thickness around 70 years of age.

**FIGURE 6:**
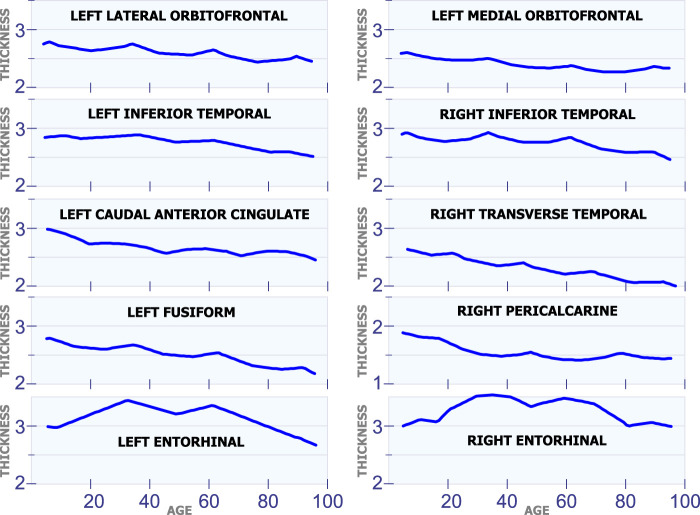
Regions with multiple changes in cortical thickness through age.

**FIGURE 7:**
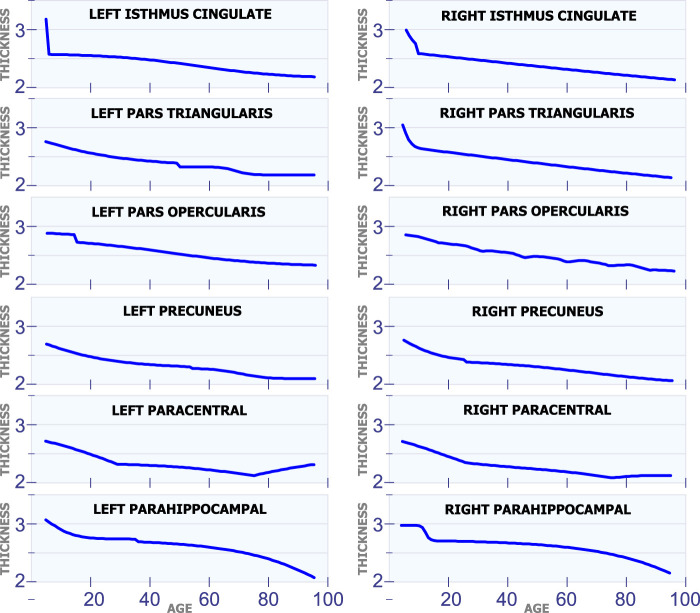
Regions with constant changes in cortical thickness.

Figure 2 shows the behavior of the models created by the artificial neural networks in twelve different regions in the brain. Each graph was built using one artificial neural network. All networks in this study had the configuration shown in Figure 1. However, each network had a different set of weights, *h* and *w*. These weights were adjusted during the training process to model one single region of the brain, and thus, discover and learn hidden patterns in the data. To build the graph, a set of uniformly distributed values for the age was applied to the input of the neural network. Then, an estimate for the cortical thickness in millimeters was produced at the output of the artificial neural network. Finally, the respective input and output values were used to build each graph in Figures 2–7.

All regions in Figure 2 exhibit a similar pattern for the changes in cortical thickness with age. Specifically, all these regions present an abrupt change in the cortical thickness speed around the age of 25 years. This abrupt change is observed by a change in the direction (line slope) of the graph for each region. As it was mentioned before, those regions of the brain with similar behavior in their cortical thickness were manually selected, and then presented in the same figure.

The first row in Figure 2 displays the cortical thickness in millimeters for the left insula and the right insula as a function of age. From this figure, it can be seen that the thickness of the left insula constantly reduces during the first 20 years of life. A similar behavior is also observed in the right insula. From age 20 to 30, the cortical thickness remains almost constant in these two regions. Then, starting at age 30, the thickness of the left and right insula starts decreasing with age at a low rate. Thus, it can be observed that both regions the left insula and the right insula exhibit a somehow similar pattern for the changes in cortical thickness with age. In the next row in Figure 2, the graphs show the cortical thickness models created using the artificial neural networks for the left superior parietal and the right superior parietal. The next row shows the models for the left precentral and right precentral. The next rows in the figure show the behavior of the cortical thickness with age in other regions of the brain; all these regions follow a similar pattern with age. However, it is important to note that the left rostral anterior cingulate and the right rostral anterior cingulate present a more abrupt change at 25 years of age than the other regions in Figure 2.

It is important to note that each artificial neural network was trained separately without using data from the same region in the other hemisphere of the brain. However, as it has been concluded by other researchers, some regions in the brain did not present the same behavior for the cortical thickness in both hemispheres. Consequently, some of the graphs in the figures do not present the results for the left hemisphere on the column on the left, and the results for the right hemisphere on the column on the right. For instance, the fifth row in Figure 2 shows the results for the left transverse temporal and the right caudal anterior cingulate.

#### 5.1.2 Changes in Cortical Thickness Around 40 years of Age

Figure 3 shows eight regions in the brain that have a special behavior in cortical thickness around 40 years of age. The first row in Figure 3 displays the cortical thickness for the left poscentral and the right poscentral. Observe that both regions exhibit a constant reduction in cortical thickness during the first 35 years of life. From 35 to 45 years of age, the cortical thickness remains almost constant in both regions. Then, starting at age 45, the cortical thickness begins to slowly decrease. The second row in Figure 3 shows the model for the left caudal middle frontal and the right caudal middle frontal. For these two regions, it can be observed a sudden and small increase in cortical thickness around age 35. In the same sense, an unexpected reduction around age 38 is present in the right lateral orbitofrontal and the right medial orbitofrontral. The last row in Figure 3 shows the behavior of the cortical thickness in the left middle temporal and the right lateral occipital. Observe that the left middle temporal exhibits an abrupt transition around are age 45, while the right lateral occipital exhibits a transition around age 32.

#### 5.1.3 Changes in Cortical Thickness Around 50 years of Age

Figure 4 shows 10 regions that have changes in cortical thickness around 50 years of age. The first row in Figure 4 displays the model for the cortical thickness in millimeters for the left inferior parietal and the right inferior parietal. In these two regions, the cortical thickness remains almost constant from age 25 to 50. Then, these regions present a slow and constant reduction in cortical starting at age 50. The graphs in the second row in Figure 4 displays the cortical thickness for the left rostral middle frontal and the right fusiform. From age 25 to 50 the cortical thickness remains approximately constant in both regions. Then, starting at age 50 there is slow a constant reduction in cortical thickness. The third row in Figure 4 shows the cortical thickness for the left cuneus and the right cuneus. Both regions present a sudden cortical thickness reduction at age 30 and 50. The fourth row in Figure 4 displays the cortical thickness behavior for the left lingual and the right lingual. An abrupt change in cortical thickness is clearly observed in both regions at age 50 years old. The last row in Figure 4 illustrates the behavior of the cortical thickness in the left pericalcarine and the right middle temporal. Notice that both regions exhibit a sudden change in cortical thickness in two different periods of life. The left pericalcarine exhibits the first change in cortical thickness around 20 years of age and the second change around 55 years of age. On the other hand, the right middle temporal has the first abrupt change at 20 years of age, while the second change is present around 45 years of age.

#### 5.1.4 Changes in Cortical Thickness Around 70 years of Age

Figure 5 shows ten regions in the human brain that present changes in cortical thickness around 70 years of age. The first row in Figure 5 illustrates these changes for the left posterior cingulate and the right posterior cingulate. These two regions exhibit a steady and non-linear reduction in cortical thickness during all stages of life. However, they have an abrupt reduction in cortical thickness around 70 years of age. All regions of the brain in Figure 5 present a very similar behavior as the ones in the first row. They have a constant and slow reduction in cortical thickness with age. They also have a sudden reduction in cortical thickness around 70 years of age.

#### 5.1.5 Regions With Changes at Multiple Ages

Figure 6 shows ten different regions that exhibit multiple cortical changes during the human lifespan. The first row in Figure 6 shows the development of the left lateral orbitofrontal and the left medial orbitofrontal. These two regions have a non-linear relation with age, and they both have a sudden increase in cortical thickness at 35 and 62 years of age. The second row in Figure 6 shows the left inferior temporal and the right inferior temporal. Again, these two regions present an abrupt increase in cortical thickness around 35 and 62 years of age. The graphs in the third row of Figure 6 include the left caudal anterior cingulate and the right transverse temporal. Both regions have inflection points at 20, 45 and 70 years of age. The graphs in the fourth row in Figure 6 include the behavior in the left fusiform and the right pericalcarine. The last row in Figure 6 shows the cortical thickness development in the left entorhinal and the right entorhinal. These are the only two regions in the brain that have very big changes in cortical thickness through the lifespan. The cortical thickness in these two regions reaches a maximum value at ages 35 and 60.

#### 5.1.6 Regions With a Constant Rate

All the regions in Figure 7 exhibit a mostly steady reduction in cortical thickness through age. The first row in Figure 7 shows the cortical thickness in millimeters for the left isthmus cingulate and the right isthmus cingulate. With the exception at the beginning of life, both of these two regions exhibit a mostly linear reduction in cortical thickness through life. The second row in Figure 7 shows the cortical thickness in millimeters for the left pars triangularis and the right pars triangularis. From the graph, it can be observed that the left pars triangularis presents an abrupt transition in cortical thickness around 50 years of age. While the right pars triangularis exhibits a linear reduction in cortical thickness for most of the human life span. The third row in Figure 7 includes the left pars opercularis and the right pars opercularis. Both of these two regions have an almost linear reduction in cortical thickness. The fourth row in Figure 7 shows the behavior of the cortical thickness in the left precuneus and the right precuneus. The left precuneus exhibits a small transition around 55 years of age, while the right precuneus exhibits a minor transition in cortical thickness around 25 years of age. The fifth row in Figure 7 shows the cortical thickness changes for the left paracentral and the right paracentral. Both of these regions have two inflection points, one at 30 of age and another at 75 years of age. The last row in Figure 7 shows the models for the left parahippocampal and the right parahippocampal. The cortical thickness for both of these regions follows a non-linear reduction through life.

### 5.2 Cortical Thickness Changes Through Life

The study of changes in cortical thickness with age is very important because it provides information about the individual. For instance, a reduction in cortical thickness has been associated with some neurodegenerative diseases (Oertel-Knöchel et al., 2015). Additionally, it has been suggested that age-related non-linear changes in cortical thickness are influenced by family income and parental education (Piccolo et al., 2016). In the same sense, Plessen et al. evaluated the connection between measures of asymmetry in cortical thickness with age, sex, and cognitive performance (Plessen et al., 2014).

In this section, we compute the derivative of the cortical thickness using Equation 3 and the models created using the artificial neural networks. The computer simulations were performed using stencils (kernels) with seven points, *N* = 7 in Equation 2. Additionally, the stencils were dynamically computed at the beginning and at the end of the lifespan to improve accuracy, see (Hassan et al., 2012). The computer simulations in Section 5.1 focused on the value and progress of the cortical thickness through different ages. On the other hand, the simulations in this section focused on the speed of the cortical thickness during the life span. Thus, when the derivative is positive, the speed is also positive and this implies that there is an increase in the cortical thickness during this part of life. When the derivative is negative the speed is also negative implying that there is a reduction in cortical thickness for that part of life. In the same sense, when the derivative is almost zero, the speed is also close to zero, and therefore, the cortical thickness does not change.

**FIGURE 8:**
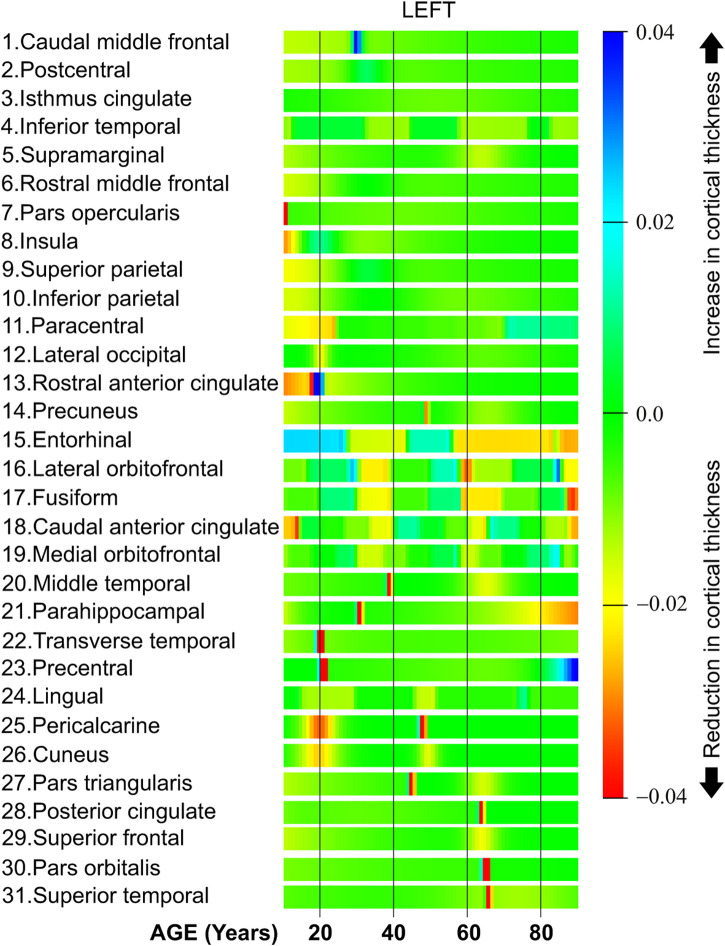
Cortical thickness derivative with respect to age, left hemisphere.

Figure 8 shows the cortical thickness derivative with respect to age. Observe that the figure includes the results only for the left hemisphere of the brain. Observe also that the results are organized in clusters, that is, those brain regions with similar derivatives are displayed next to each other. The thickness derivative is represented using the color scale displayed on the right part of Figure 8. Starting at the top of the scale, the blue dark color is used to display a significant increase in cortical thickness. In the middle of the scale, the green color is used to indicate no changes in cortical thickness, 0.0. At the bottom of the scale, the red color is used to indicate an important reduction in cortical thickness.

Row one in Figure 8 shows the derivative for the caudal middle frontal. As it can be seen this band is mostly green with a blue band around 30. Therefore, this region exhibits a constant derivative with an abrupt increase in the cortical thickness speed around 30 years of age. The bands from row two (postcentral) to row six (rostral middle frontal) in Figure 8 are mostly green with some soft yellow zones. Thus, these brain regions exhibit an almost constant cortical thickness derivative during the lifespan. From row seven in Figure 8 (pars opercularis) to row twelve (lateral occipital) all these bands have red and yellow zones at the beginning of life. Thus, these brain regions lose cortical thickness at high speed around the first 20 years of age. Row 13 in Figure 8 shows the behavior of the rostral anterior cingulate. There are red, yellow and blue color bands in the first 20 years of life. This implies that the cortical thickness speed considerably changes during the first 2 decades of life. From row 15 (entorhinal) to row 21 (parahippocampal), all these brain regions present different cortical thickness speeds at diverse parts of life. Both the transverse temporal in row 22 and the precentral in row 23 have a red zone around 20 years of age. This implies that the human brain presents a period with great reductions in cortical thickness for these two regions at age 20.

All regions from row 24 (lingual) to row 26 (cuneus) exhibit a red or yellow band around 20 and 50 years. This means that during this age, the derivative is negative, and therefore, the cortical thickness is quickly reduced during these two parts of life. The last regions in Figure 8 starting in row 28 (posterior cingulate) have a red band around 65 years of age. Thus, these regions exhibit a fast reduction in cortical thickness at 65 years.

**FIGURE 9:**
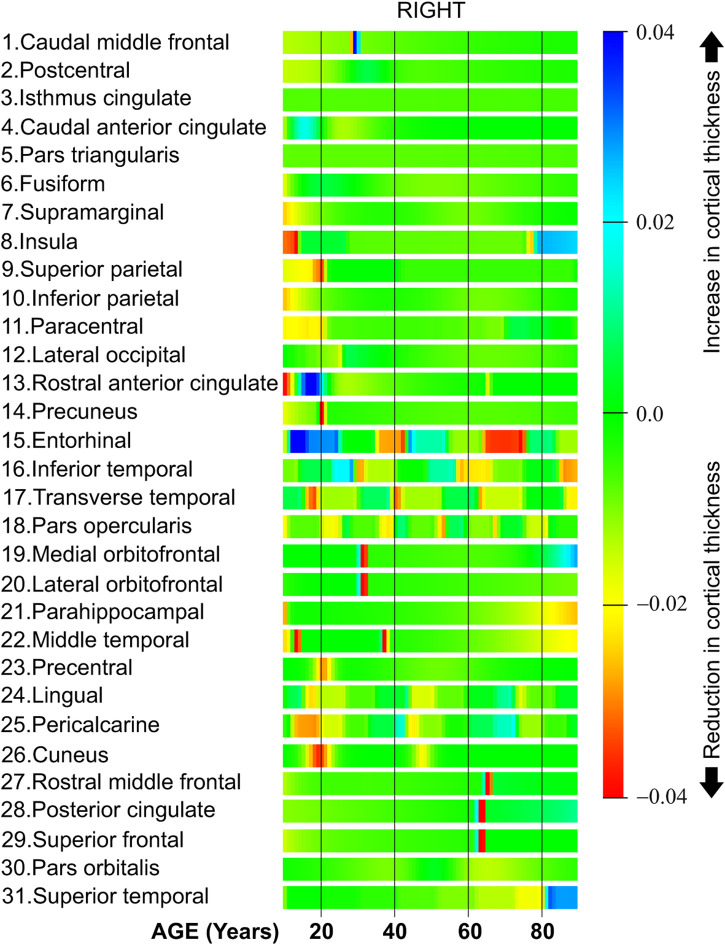
Cortical thickness derivative with respect to age, right hemisphere.

Figure 9 shows the derivative of the cortical thickness for the right hemisphere. The regions in Figure 9 are organized in clusters as in the regions in the left hemisphere.

The first six regions in Figure 9, from caudal middle frontal to fusiform, have a constant cortical thickness speed for most of the life. Regions from row 7 (supramarginal) to 11 (paracentral) present a high cortical thickness reduction during the first 20 years of life. The regions located in the cluster in the middle of Figure 9, from row 15 (entorhinal) to row 18 (pars opercularis), have several abrupt changes in the cortical thickness speed at different parts of life. Both the medial orbitofrontal in row 19 and the lateral orbitofrontal in row 20 have a negative cortical thickness speed around 33 years of age. The regions from row 24 (lingual) to 26 (cuneus) have a negative cortical thickness speed around 20 and 50 years of age. Finally, the regions from row 27 (rostral middle frontal) to 31 (superior temporal) present a negative cortical thickness speed around 70 years of age.

Tables 3–5 show some of the main results from this study. The first row in Table 3 indicates that the left pericalcarine is the region with the lowest cortical thickness throughout all life. As it can be seen from the third column in Table 3, the right entorhinal is the region with the highest thickness throughout all life, after 40 years of age, and after 60 years of age. However, the value in the second column in the last row in Table 3 indicates that the right pericalcarine is the region with the lowest thickness after 60 years of age.

**TABLE 3:** Modeling of the cortical thickness through life.

	Lowest thickness	Highest thickness
Through all life	Left pericalcarine	Right entorhinal
age ≥40	Left pericalcarine	Right entorhinal
age ≥60	Right pericalcarine	Right entorhinal

Table 4 shows the variability of the cortical thickness. Through all life, the region with the lowest variability is the right caudal anterior cingulate, and the region with the highest variability is the left parahippocampal. For a person 40 years and older, the region with the lowest variability is again the right caudal anterior cingulate, while the region with the highest variability is the right transverse temporal. For a person 60 years and older, the left enthorhinal is the region with the highest variability, and the left pericalcarine is the region with the lowest variability.

**TABLE 4:** Variability of the cortical thickness through life.

	Lowest variability	Highest variability
Through all life	Right caudal anterior cingulate	Left parahippocampal
age ≥40	Right caudal anterior cingulate	Right transverse temporal
age ≥60	Left pericalcarine	Left enthorhinal

Table 5 measures the linearity of the cortical thickness with age. Throughout life, the left lateral occipital is the region that exhibits the highest linearity. For an age of 40 years and older, the right isthmus cingulate is the region with the highest linearity. For an age of 60 years and older, the left pericalcarine is the region with the highest linearity. In this sense, the cortical thickness in those regions in the third column of Table 5 can be estimated using a simple linear model. On the other hand, the cortical thickness of those regions in the second column of Table 5 cannot be accurately predicted using a simple linear model. In summary, the models created with artificial neural networks adapt to the patterns in the data. Therefore, the performance of a neural network model or a linear model is very similar in those regions that exhibit a linear tendency in its cortical thickness with time. For those regions that have a linear behavior, the mean squared error was 0.016 for both models. However, the performance of the neural network models was better than the performance of linear models in those regions with complex patterns through age. For those regions that do not have a linear behavior with time, the mean squared error for the neural network models was 0.03 while the mean squared error for the linear models was 3.0.

**TABLE 5:** Linearity of the cortical thickness through life.

	Lowest linearity	Highest linearity
Through all life	Right entorhinal	Left lateral occipital
age ≥40	Right entorhinal	Right isthmus cingulate
age ≥60	Left lateral orbitofrontal	Left pericalcarine

In this publication, we propose the use of artificial neural networks to model the thickness of the cortical thickness through life for different regions in the brain. Once the neural networks are trained, it is possible to validate the performance of the model using new datasets. One important feature of artificial neural networks is their capacity to generalize. This means that a neural network has been trained, it should be able to predict the cortical thickness of data that the network has not seen before (Masters, 2015). Future work may include the study on how to utilize the artificial neural network models to understand various cognitive functions through life.

## 6 Conclusion

This work analyzes the progress of the cortical thickness with age using Artificial Intelligence. A set of artificial neural networks was trained and validated using a dataset with information from 1,100 healthy individuals. Each neural network was designed to model one single region in the human brain. Thus, 31 artificial neural networks were created to model the cortical thickness in each region in the left hemisphere of the brain. Similarly, 31 networks were created to model the cortical thickness for the regions in the right hemisphere. Furthermore, computer simulations were used to adjust the number of neurons in the hidden layer of the artificial neural networks, and thus, obtain the best model given the amount of data available.

The models created by the artificial neural networks were, then, organized in clusters. Each cluster included those regions that followed a similar pattern for the cortical thickness through age. The results from the computer simulations show that the models allow the detection of abrupt changes in cortical thickness. The simulations also provide an age estimate of when these changes may happen.

Additionally, the neural networks were used with numerical differentiation techniques to estimate the derivative of the cortical thickness with respect to age. Dynamic stencils were used to improve the accuracy of the derivative at the beginning and the end of life. Then, color bands were created to display the speed of the cortical thickness. A color scale was designed to locate and visualize those parts of life with a positive or a negative speed. A positive speed is obtained when there is an increase in cortical thickness. On the other hand, a negative speed is present when there is a reduction in cortical thickness during that part of life. Therefore, the color bands allowed the detection of those parts of life with a reduction or an increase in cortical thickness. Finally, these graphs were organized in clusters. Each cluster included those regions with similar behavior through life.

After examining the results, it was concluded that some regions in the left hemisphere do not present the same progress with age as the counterpart regions in the right hemisphere. Some regions in the brain exhibit very particular patterns in their cortical thickness; one of these regions is the entorhinal. One advantage of the methodology proposed in this paper is that the models created using the artificial neural networks do not assume a linear or non-linear model. Instead, the artificial neural network is capable of dynamically adapt to the required complexity of each region in the human brain. Additionally, artificial neural networks are insensitive to noise present in the data and learn the patterns relevant to the specific application. Most importantly, neural networks are capable of generalizing, that is, they are able to predict patterns that are present in other datasets that were not used for training.

## Data Availability

Publicly available datasets were analyzed in this study. This data can be found here: OASIS: http://oasis-brains.org/ IXI Dataset https://brain-development.org/ixi-dataset/ NKI-RS http://fcon_1000.projects.nitrc.org/indi/enhanced/.

## References

[B1] Alpaydin E. (2016). Machine Learning: The New AI (The MIT Press Essential Knowledge Series). Massachusetts, London, England: The MIT Press Cambridge.

[B2] Chen Z. J. He Y. Rosa-Neto P. Gong G. Evans A. C. (2011). Age-Related Alterations in the Modular Organization of Structural Cortical Network by Using Cortical Thickness from MRI. NeuroImage. 56, 235–245. 10.1016/j.neuroimage.2011.01.010 21238595

[B3] Churchwell J. C. Yurgelun-Todd D. A. (2013). Age-Related Changes in Insula Cortical Thickness and Impulsivity: Significance for Emotional Development and Decision-Making. Developmental Cogn. Neurosci. 6, 80–86. 10.1016/j.dcn.2013.07.001 PMC6987805 23921157

[B4] Fischl B. (2012). FreeSurfer. NeuroImage. 62, 774–781. 10.1016/j.neuroimage.2012.01.021 22248573 PMC3685476

[B5] Fjell A. M. Westlye L. T. Grydeland H. Amlien I. Espeseth T. Reinvang I. (2014). Accelerating Cortical Thinning: Unique to Dementia or Universal in Aging?. Cereb. Cortex. 24, 919–934. 10.1093/cercor/bhs379 23236213 PMC3948495

[B6] Fouche J.-P. du Plessis S. Hattingh C. Roos A. Lochner C. Soriano-Mas C. (2017). Cortical Thickness in Obsessive-Compulsive Disorder: Multisite Mega-Analysis of 780 Brain Scans From Six Centres. Br. J. Psychiatry. 210, 67–74. 10.1192/bjp.bp.115.164020 27198485

[B7] Goodfellow I. Bengio Y. Courville A. (2016). Deep Learning (Adaptive Computation and Machine Learning Series). Massachusetts, London, England: The MIT Press Cambridge.

[B8] Hassan H. Z. Mohamad A. A. Atteia G. E. (2012). An Algorithm for the Finite Difference Approximation of Derivatives With Arbitrary Degree and Order of Accuracy. J. Comput. Appl. Mathematics. 236, 2622–2631. 10.1016/j.cam.2011.12.019

[B9] Information eXtraction Images (2019). IXI Dataset, Biomedical Image Analysis Group, Information eXtraction from Images. London: Imperial College London, South Kensington Campus. Available at: https://brain-development.org/ixi-dataset/ (Accessed December 4, 2019).

[B10] Jin B. Jing Z. Zhao H. (2017). Incremental and Decremental Extreme Learning Machine Based on Generalized Inverse. IEEE Access. 5, 20852–20865. 10.1109/access.2017.2758645

[B11] Jordan M. I. Mitchell T. M. (2015). Machine Learning: Trends, Perspectives, and Prospects. Science. 349, 255–260. 10.1126/science.aaa8415 26185243

[B12] Kelleher J. D. Namee B. M. D’Arcy A. (2015). Fundamentals of Machine Learning for Predictive Data Analytics: Algorithms, Worked Examples, and Case Studies. Cambridge, MA: Massachusetts Institute of Technology.

[B13] Klein A. Tourville J. (2012). 101 Labeled Brain Images and a Consistent Human Cortical Labeling Protocol. Front. Neurosci. 6, 171–212. 10.3389/fnins.2012.00171 23227001 PMC3514540

[B14] Landman B. A. Huang A. J. Gifford A. Vikram D. S. Lim I. A. L. Farrell J. A. D. (2011). Multi-Parametric Neuroimaging Reproducibility: A 3-T Resource Study. NeuroImage. 54, 2854–2866. 10.1016/j.neuroimage.2010.11.047 21094686 PMC3020263

[B15] Ledesma S. Ibarra-Manzano M. A. Garcia-Hernandez M. G. Almanza-Ojeda D. L. (2017). Neural Lab a Simulator for Artificial Neural Networks. London, United Kingdom: IEEE Computing Conference, 716–721.

[B16] Lemaitre H. Goldman A. L. Sambataro F. Verchinski B. A. Meyer-Lindenberg A. Weinberger D. R. (2012). Normal Age-Related Brain Morphometric Changes: Nonuniformity Across Cortical Thickness, Surface Area and Gray Matter Volume? Neurobiol. Aging. 33, 617–619. 10.1016/j.neurobiolaging.2010.07.013 PMC3026893 20739099

[B17] Marcus D. S. Wang T. H. Parker J. Csernansky J. G. Morris J. C. Buckner R. L. (2007). Open Access Series of Imaging Studies (OASIS): Cross-Sectional MRI Data in Young, Middle Aged, Nondemented, and Demented Older Adults. J. Cogn. Neurosci. 19, 1498–1507. 10.1162/jocn.2007.19.9.1498 17714011

[B18] Marsland S. (2015). Machine Learning: An Algorithmic Perspective. Second Edition. Boca Raton, London, New York: Chapman & Hall/Crc Machine Learning & Pattern Recognition, CRC Press, Taylor & Francis Group.

[B19] Masters T. (2015). Deep Belief Nets in C++ and CUDA, Restricted Boltzmann Machines. Lexington, KY, USA: Masters.

[B20] McCarthy C. S. Ramprashad A. Thompson C. Botti J. A. Coman I. L. Kates W. R. (2015). A Comparison of FreeSurfer-Generated Data With and Without Manual Intervention. Front. Neurosci. 9, 379–418. 10.3389/fnins.2015.00379 26539075 PMC4612506

[B21] McGinnis S. M. Brickhouse M. Pascual B. Dickerson B. C. (2011). Age-Related Changes in the Thickness of Cortical Zones in Humans. Brain Topogr. 24, 279–291. 10.1007/s10548-011-0198-6 21842406 PMC3600370

[B22] Oertel-Knöchel V. Reuter J. Reinke B. Marbach K. Feddern R. Alves G. (2015). Association Between Age of Disease-Onset, Cognitive Performance and Cortical Thickness in Bipolar Disorders. J. Affective Disord. 174, 627–635. 10.1016/j.jad.2014.10.060 25577157

[B23] Piccolo L. R. Merz E. C. He X. Sowell E. R. Noble K. G. (2016). Age-Related Differences in Cortical Thickness Vary by Socioeconomic Status. PLOS one. 11, e0162511–18. 10.1371/journal.pone.0162511 27644039 PMC5028041

[B24] Plessen K. J. Hugdahl K. Bansal R. Hao X. Peterson B. S. (2014). Sex, Age, and Cognitive Correlates of Asymmetries in Thickness of the Cortical Mantle Across the Life Span. J. Neurosci. 34, 6294–6302. 10.1523/jneurosci.3692-13.2014 24790200 PMC4004815

[B25] Razlighi Q. R. Habeck C. Barulli D. Stern Y. (2017). Cognitive Neuroscience Neuroimaging Repository for the Adult Lifespan. NeuroImage. 144, 294–298. 10.1016/j.neuroimage.2015.08.037 26311605 PMC4766063

[B26] Russell S. Norvig P. (2020). Artificial Intelligence: A Modern Approach. 4th Edition. Hoboken, NJ: Pearson International.

[B27] Salat D. H. Buckner R. L. Snyder A. Z. Greve D. N. Desikan R. S. R. Busa E. (2004). Thinning of the Cerebral Cortex in Aging. Cereb. Cortex. 14, 721–730. 10.1093/cercor/bhh032 15054051

[B28] Scott M. L. J. Bromiley P. A. Thacker N. A. Hutchinson C. E. Jackson A. (2009). A Fast, Model-Independent Method for Cerebral Cortical Thickness Estimation Using MRI. Med. Image Anal. 13, 269–285. 10.1016/j.media.2008.10.006 19068276

[B29] Sowell E. R. Peterson B. S. Kan E. Woods R. P. Yoshii J. Bansal R. (2007). Sex Differences in Cortical Thickness Mapped in 176 Healthy Individuals Between 7 and 87 Years of Age. Cereb. Cortex. 17, 1550–1560. 10.1093/cercor/bhl066 16945978 PMC2329809

[B30] Steffener J. Habeck C. O'Shea D. Razlighi Q. Bherer L. Stern Y. (2016). Differences Between Chronological and Brain Age Are Related to Education and Self-Reported Physical Activity. Neurobiol. Aging. 40, 138–144. 10.1016/j.neurobiolaging.2016.01.014 26973113 PMC4792330

[B31] Tamnes C. K. Østby Y. Fjell A. M. Westlye L. T. Due-Tønnessen P. Walhovd K. B. (2010). Brain Maturation in Adolescence and Young Adulthood: Regional Age-Related Changes in Cortical Thickness and White Matter Volume and Microstructure. Cereb. Cortex. 20, 534–548. 10.1093/cercor/bhp118 19520764

[B32] Tustison N. J. Cook P. A. Klein A. Song G. Das S. R. Duda J. T. (2014). Large-Scale Evaluation of ANTs and FreeSurfer Cortical Thickness Measurements. NeuroImage. 99, 166–179. 10.1016/j.neuroimage.2014.05.044 24879923

[B33] Wierenga L. M. Langen M. Oranje B. Durston S. (2014). Unique Developmental Trajectories of Cortical Thickness and Surface Area. NeuroImage. 87, 120–126. 10.1016/j.neuroimage.2013.11.010 24246495

[B34] Winkler A. M. Greve D. N. Bjuland K. J. Nichols T. E. Sabuncu M. R. Håberg A. K. (2018). Joint Analysis of Cortical Area and Thickness as a Replacement for the Analysis of the Volume of the Cerebral Cortex. Cereb. Cortex. 28, 738–749. 10.1093/cercor/bhx308 29190325 PMC5972607

